# Activation of the Kynurenine Pathway in Human Malignancies Can Be Suppressed by the Cyclin-Dependent Kinase Inhibitor Dinaciclib

**DOI:** 10.3389/fimmu.2020.00055

**Published:** 2020-02-14

**Authors:** Christin Riess, Björn Schneider, Hanna Kehnscherper, Julia Gesche, Nina Irmscher, Fatemeh Shokraie, Carl Friedrich Classen, Elisa Wirthgen, Grazyna Domanska, Annette Zimpfer, Daniel Strüder, Christian Junghanss, Claudia Maletzki

**Affiliations:** ^1^University Children's Hospital, Rostock University Medical Centre, Rostock, Germany; ^2^Institute for Medical Microbiology, Virology, and Hygiene, Rostock University Medical Centre, Rostock, Germany; ^3^Medical Clinic III - Hematology, Oncology, Palliative Care, Department of Internal Medicine, Rostock University Medical Center, Rostock, Germany; ^4^Institute of Pathology, Rostock University Medical Center, University of Rostock, Rostock, Germany; ^5^Institute of Immunology and Transfusion Medicine, University of Greifswald, Greifswald, Germany; ^6^Department of Otorhinolaryngology, Head and Neck Surgery “Otto Koerner”, Rostock University Medical Center, Rostock, Germany

**Keywords:** targeted therapy, solid tumor models, tryptophan metabolites, IDO1, chemotherapy

## Abstract

Indoleamine 2,3-dioxygenase (IDO) and tryptophan 2,3-dioxygenase (TDO2) are the key enzymes of tryptophan (TRP) metabolism in the kynurenine pathway (KP). Both enzymes function as indicators of immunosuppression and poor survival in cancer patients. Direct or indirect targeting of either of these substances seems thus reasonable to improve therapy options for patients. In this study, glioblastoma multiforme (GBM) as well as head and neck squamous cell carcinomas (HNSCC) were examined because of their different mechanisms of spontaneous and treatment-induced immune escape. Effects on gene expression and protein levels were examined. Accompanying assessment of TRP metabolites from treated GBM cell culture supernatants was conducted. Our results show a heterogeneous and inversely correlated expression profile of TRP-metabolizing genes among GBM and HNSCC cells, with low, but inducible *IDO1* expression upon IFNγ treatment. *TDO2* expression was higher in GBM cells, while genes encoding kynurenine aminotransferases were mainly confined to HNSCC cells. These data indicate that the KP is active in both entities, with however different enzymes involved in TRP catabolism. Upon treatment with Temozolomide, the standard of care for GBM patients, *IDO1* was upregulated. Comparable, although less pronounced effects were seen in HNSCC upon Cetuximab and conventional drugs (i.e., 5-fluorouracil, Gemcitabine). Here, *IDO1* and additional genes of the KP (*KYAT1, KYAT2*, and *KMO*) were induced. Vice versa, the novel yet experimental cyclin-dependent kinase inhibitor Dinaciclib suppressed KP in both entities. Our comprehensive data imply inhibition of the TRP catabolism by Dinaciclib, while conventional chemotherapeutics tend to activate this pathway. These data point to limitations of conventional therapy and highlight the potential of targeted therapies to interfere with the cells' metabolism more than anticipated.

## Introduction

Tumor cells release immunosuppressive factors that shape a tolerogenic environment and enable progression and invasion. Indoleamine 2,3-dioxygenase (IDO1) is an intracellular monomeric, immune-checkpoint molecule that degrades the essential amino acid l-tryptophan along the kynurenine pathway (KP) (1, 2). Like other immune checkpoints, including programmed cell death protein 1 and cytotoxic T-lymphocyte-associated protein 4, IDO suppresses the hosts' antitumor immunity by inducing apoptosis in T- and natural killer cells (3). As a direct consequence of this, many cancer and cancer-associated cells express *IDO1* (mesenchymal stromal cells, myeloid-derived suppressor cells, dendritic cells, endothelial cells, tumor-associated macrophages, and fibroblasts) (3–6). *IDO1* is influenced by interferon-γ (IFNγ) (7–9), nitric oxide (10), pro- [interleukin (IL)-1β, tumor necrosis factor α] and anti-inflammatory (IL4, IL10, transforming growth factor β) cytokines. *IDO1* activity inhibits T-cell activation and proliferation and even mediates regulatory T-cell recruitment to the tumor microenvironment, provoking local immune tolerance. In head and neck squamous cell carcinomas (HNSCCs), *IDO1* inversely correlates with programmed cell death protein ligand 1, which constitutes an important prognostic biomarker for immune-checkpoint inhibition (11). The increased IDO1 activation decreases intratumoral TRP levels, resulting in tumor starvation and increase in kynurenine (KYN) metabolites (which are toxic to lymphocytes) (12). This immune exhaustion may be further boosted by conventional chemotherapeutics, leading to decreased efficacy. Therefore, *IDO1* overexpression in the tumor microenvironment intimately impairs patients' outcome and may serve as a future prognostic predictor and drug target (13–18).

In the KP, most studies focused on IDO1 because this molecule is amenable to pharmacological intervention (19–22), and a couple of specific and global IDO inhibitors [including natural compounds (17, 23, 24)] already entered clinical trials, mostly reporting safe application and efficacy (stable disease at best outcome) (25). Current trials are evaluating the efficacy of IDO1 inhibitors in combination with chemotherapy, radiotherapy, and other immunotherapies including cytotoxic T-lymphocyte-associated protein 4 blockade (11, 22). The latter is based on the observation of an enhanced lytic ability of tumor-antigen-specific T cells upon IDO1 inhibition and decreased numbers of local immunosuppressive cells such as regulatory T cells and myeloid-derived suppressor cells (20, 26). The efficacy and toxicity data from recent clinical trials with IDO1 inhibitors is reviewed in Yentz and Smith (27). In most cases, however, overall survival was not significantly improved, leaving the future role for this combination therapy in question (28). More key enzymes are involved in TRP metabolism: tryptophan 2.3-dioxygenase (TDO2), a member of the oxidoreductases family, catalyzes the same initial step of the KP as IDO1 (2). Thus, TDO2 has been shown to be constitutively and highly expressed in various cancer cells such as malignant glioma and HNSCC (29, 30). More importantly, TDO2 also has immunomodulatory functions by promoting immune tolerance. This, in turn, promotes survival, growth, invasion, and metastasis and decreases patients' survival (just like *IDO1*) (13, 22, 31, 32).

In this study, we performed a comprehensive analysis on the expression status of genes belonging to the KP. HNSCC and glioblastoma multiforme (GBM) were picked as prime examples for different spontaneous and treatment-induced immune escape mechanisms. Therefore, expression changes were determined under standard and targeted therapy, and results were compared among each other.

## Materials and Methods

### Tumor Cell Lines and Culture Conditions

Patient-derived GBM cell lines (*N* = 13; HROG02, HROG04, HROG05, HROG06, HROG10, HROG15, HROG24, HROG36, HROG38, HROG52, HROG63, HROG73, HROG75) and HNSCC cell lines (*N* = 6; FADU, Detroit-562, Cal-33, PE/CA/PJ-15, UT-SCC-14, UT-SCC-15) were either established and basically characterized in our lab or originally obtained from the German collection of cell cultures (DSMZ; Braunschweig, Germany). UT-SCC14 and UT-SCC15 cells were kindly provided by Prof. R. Grenman [University of Turku, Finland (33)]. All cells were routinely cultured in our lab and maintained in full medium: Dulbecco's modified Eagle Medium/HamsF12 supplemented with 10% fetal calf serum, glutamine (2 mmol/L), and antibiotics (medium and supplements were purchased from PAA, Cölbe, Germany). For functional analysis, cell lines from each tumor entity were chosen, and all subsequent experiments were performed with these lines only.

### IFNγ Stimulation

Cells were cultured in six-well plates or ibidi chamber slides, incubated overnight and treated with IFNγ (50 ng/ml, Immunotools, Friesoythe, Germany) for 24 and 72 h, respectively. Thereafter, cells were harvested and further processed.

### Cytostatic Drugs and Targeted Substance

Cytostatics used in this study included 5-fluorouracil (5-FU) (2.5 μM), Cisplatin (0.2 μM), Gemcitabine (0.0002 μM), and Cetuximab (0.34 μM) for HNSCC, as well as Temozolomide (10 μM, TMZ) for GBM (pharmacy of the University Hospital Rostock). CDKi Dinaciclib (10 or 100 nM) was used as experimental targeted drug. All substances were used in doses below the IC_50_ as determined before.

### Apoptosis/Necrosis Assay

A Yo-Pro-1/PI-based assay for discriminating early apoptotic, late apoptotic, and necrotic cells was applied as described before (34).

### Hemolysis Assay

Hemolytic activity of Dinaciclib was determined by hemoglobin release from whole blood cells after 2 h of incubation. Briefly, whole blood of healthy donors (*N* = 5) was seeded in 96-well plates and treated with increasing Dinaciclib doses (ranging from 1, 5, and 10 μM). Negative controls were left untreated, and positive controls (=maximum lysis) were treated with 1% sodium dodecyl sulfate. Following the incubation period, cell-free supernatants were transferred into a new 96-well plate, and absorption was measured on a plate reader at 560 nm (reference wave length, 750 nm). Hemolytic activity was quantified according to the following formula and corrected for spontaneous hemolysis (=untreated controls):

$$\begin{array}{l} \%\text{Hemolysis}=(({\text{OD}}_{560\text{nm}}\text{sample}\\ -{\text{OD}}_{560\text{nm}}\text{buffer})/{\text{OD}}_{560\text{nm}}\text{max}-{\text{OD}}_{560\text{nm}}\text{buffer})\times 100 \end{array}$$

In addition, peripheral blood mononuclear cells' (PBMC) viability (*N* = 5) were determined by Calcein AM staining. This was done upon 24 h incubation at the above-mentioned doses. Fluorescence measurement and quantification were done as described (34).

### IDO1 Immunofluorescence

Tumor cells were treated with 50 ng/ml of IFNγ (Immunotools), TMZ, Cetuximab, or Dinaciclib for 24 h in chamber slides, respectively. Cells were washed with phosphate-buffered saline, fixed in 4% paraformaldehyde w/o methanol (Thermo Scientific, Darmstadt, Germany) for 20 min, washed again, followed by cell permeabilization in 0.3% Triton X−100/5% normal bovine serum in phosphate-buffered saline for 60 min. Cells were then incubated overnight at 4°C in monoclonal rabbit IDO1 primary antibody (1:100; Cell Signaling Technology, Frankfurt/Main, Germany). Cells were washed, labeled with fluorochrome-conjugated secondary antibody using goat antirabbit secondary antibody (1:250, Boster Biological Technology, Pleasanton CA, USA), and incubated in the dark for 2 h. Cell nuclei were stained with 4′,6-diamidino-2-phenylindole (DAPI), and cells were analyzed with a Zeiss LSM-780 Confocal Laser Microscope (Zeiss, Jena, Germany). Quantification of staining intensity was done using the ImageJ software. Therefore, channels were split into red, green, and blue. Subsequently, integrated density profiles of the same size were measured in the green channel.

### IDO1 Immunohistochemistry on Patients' Tumor Samples

Primary antibody against IDO1 (rabbit IgG, clone D5J4E, Cell Signaling Technology, dilution 1:200) was used. All samples were pretreated for 20 min at 97°C and pH 6.9. Standard immunoperoxidase technique was applied using an automated immunostainer (DAKO link) with diaminobenzidine as chromogen. IDO1 expression was defined as cytoplasmatic and membranous staining in >1% inflammatory cells.

### Quantification of Tryptophan, Kynurenine, and Kynurenic Acid in Cell Culture Supernatant by Liquid Chromatography Tandem Mass Spectrometry System

The basis for the measurement was the method of Fuertig et al. which was adapted to the system used here (35).

#### Sample Preparation

Cell culture supernatant was mixed 1:1 with internal standards [10 μM D5-kynurenic acid (Buchem BV, Apeldoorn, Netherlands), 10 μM D5-phenylalanine (Cambridge Isotope Laboratories, Inc. Andover, MA, United States), 5 μM D4-kynurenine (Cambridge Isotope Laboratories), 10 μM D5-tryptophan (Sigma Aldrich, Hamburg, Germany), 10 μM D3-quinolinic acid (Buchem BV), 5.5 nM 15N5-8-hydroxy-2-deoxyguanosine (Cambridge Isotope Laboratories)], and with 10 μl of mobile phase (0.4% formic acid, 1% acetonitrile in water). Reagents were gently shaken on a mixer, and 150 μl of ice-cold methanol was added. Samples were incubated overnight at −20°C to allow protein precipitation. On the following day, samples were centrifugated at 0°C and 18,000×*g* for 15 min. Supernatants were transferred to a new tube, and the liquid phase was removed by evaporation at 30°C among vacuum. Solid samples were stored until measurement at −20°C. Afterwards, dried extracts were reconstituted in 100 μl of acidified mobile phase. Samples were incubated at 40°C (1 h), centrifuged (4°C, 18,000×*g*, 5 min), and clear supernatant (100 μl) was transferred onto a 96-well plate.

#### Liquid Chromatography Tandem Mass Spectrometry

Measurements were performed on an AB Sciex 5500 QTrap™ mass spectrometer (AB SCIEX, Darmstadt, Germany) with electrospray ionization in positive mode combined with a high-performance liquid chromatography system (Agilent 1260 Infinity Binary LC, Santa Clara, United States) including a degasser unit, column oven, autosampler, and a binary pump. Twenty microliters of the supernatant was injected and separated using a VisionHT C18 column (100 × 2.1 mm; particle size, 3 μm; Grace, MD, United States). To prevent contamination, a precolumn (VisionHT C18, Guard 5 × 2 mm) was used additionally. The temperature of the column oven was set at 15°C. The flowrate was set to 0.4 ml/min, and the sample was separated in a total run time of 11 min using solution A (water + 0.1% formic acid + 0.01% trifluoroacetic acid) and solution B (MeOH + 0.1% formic acid + 0.01% trifluoroacetic acid) with the following gradient: 0–2.8 min, 97% A, 3% B; 2.8–3.3 min, 70% A, 30% B; 3.3–4.4, 40% A, 60% B; 4.5–5.0 min, 40% A, 60% B; 5.0–5.5, 5% A, 95% B; 5.5–6.9 min, 5% A, 95% B; 6.9–7.0 min, 97% A, 3% B; 7.0–11.0 min, 97% A, 3% B.

The eluate between 0.5 and 9 min was introduced into the mass spectrometer and analyzed in MRM mode. The ion spray voltage (IS) was 4,000 V, the curtain gas flow was 40.0 psi, and the ion source temperature were set at 550°C.

Internal standards were used for metabolite quantification (Table 1). Data analysis, including peak integration and concentration determination, was performed with Analyst software (Version 1.5.1, AB Sciex, Darmstadt, Germany).

**Table 1:** Internal standards.

**Analyte**	**Q1 mass (m/z)**	**Q3 mass (m/z)**	**CE (V)**	**DP (V)**
Tryptophan	205.1	118.0	28.0	39.0
d5-Tryptophan	210.1	122.1	37.0	31.0
Kynurenine	209.1	94.1	19.6	41.0
d4-Kynurenine	213.1	140.1	21.0	39.0
Kynurenic acid	190.1	162.0	24.0	65.0
d5-Kynurenic acid	195.1	167.1	24.0	65.0

### RNA Isolation, cDNA Synthesis, and Quantitative Real-Time PCR

Total RNA was isolated with RNeasy Mini Kit (Qiagen, Hilden, Germany) according to the manufacturers' instructions. RNA was reverse transcribed into complementary DNA (cDNA) from 1 μg RNA using 1 μl dNTP mix (10 mM), oligo (dT)15 primer (50 ng/μl), 1 μl reverse transcriptase (100 U), and 4 μl 5× reverse transcription buffer complete (all purchased from Bioron GmbH, Ludwigshafen, Germany). Final reaction volume was 20 μl (filled with RNAse free water). cDNA synthesis conditions were as follows: 70°C for 10 min, 45°C for 120 min, and 70°C for 10 min. Target cDNA levels of human cell lines were analyzed by quantitative real-time PCR using TaqMan Universal PCR Master Mix and self-designed TaqMan gene expression assays either labeled with 6-FAM-3′ BHQ-1 or 5′ HEX−3′ BHQ-1 to be used as duplex: *IDO1, TDO2, KMO, HAAO, KYAT1/2/3/4, KYNU, QPRT*, and *GAPDH* or ß*-actin* were used as housekeeping genes. Reaction was performed in the light cycler Viia7 (Applied Biosystems, Foster City, USA) with the following PCR conditions: 95°C for 10 min, 40 cycles of 15 s at 95°C, and 1 min at 60°C. All reactions were run in triplicates. The messenger RNA (mRNA) levels of target genes were normalized to *GAPDH/*β*-actin*. Reactions were performed in triplicate wells and repeated four times. The general expression level of each sample was considered by calculating 2^−ΔCT^ (ΔCt = Ct_target_ – Ct_Housekeeping genes_).

### Statistical Analysis

All values are reported as mean ± SD. After proving the assumption of normality, differences between controls and treated cells were determined using the unpaired Student's *t*-test. If normality failed, the non-parametric Mann-Whitney *U*-test was applied. Statistical evaluation was performed using GraphPad PRISM software, version 5.02 (GraphPad Software, San Diego, CA, USA). In case of multiple comparisons, two- or one-way ANOVA on ranks (Bonferroni's multiple comparison test) was used. The criterion for significance was taken to be *p* < 0.05.

## Results

### Basal IDO1 and Related Genes in GBM and HNSCC Cell Lines

**Figure 1:**
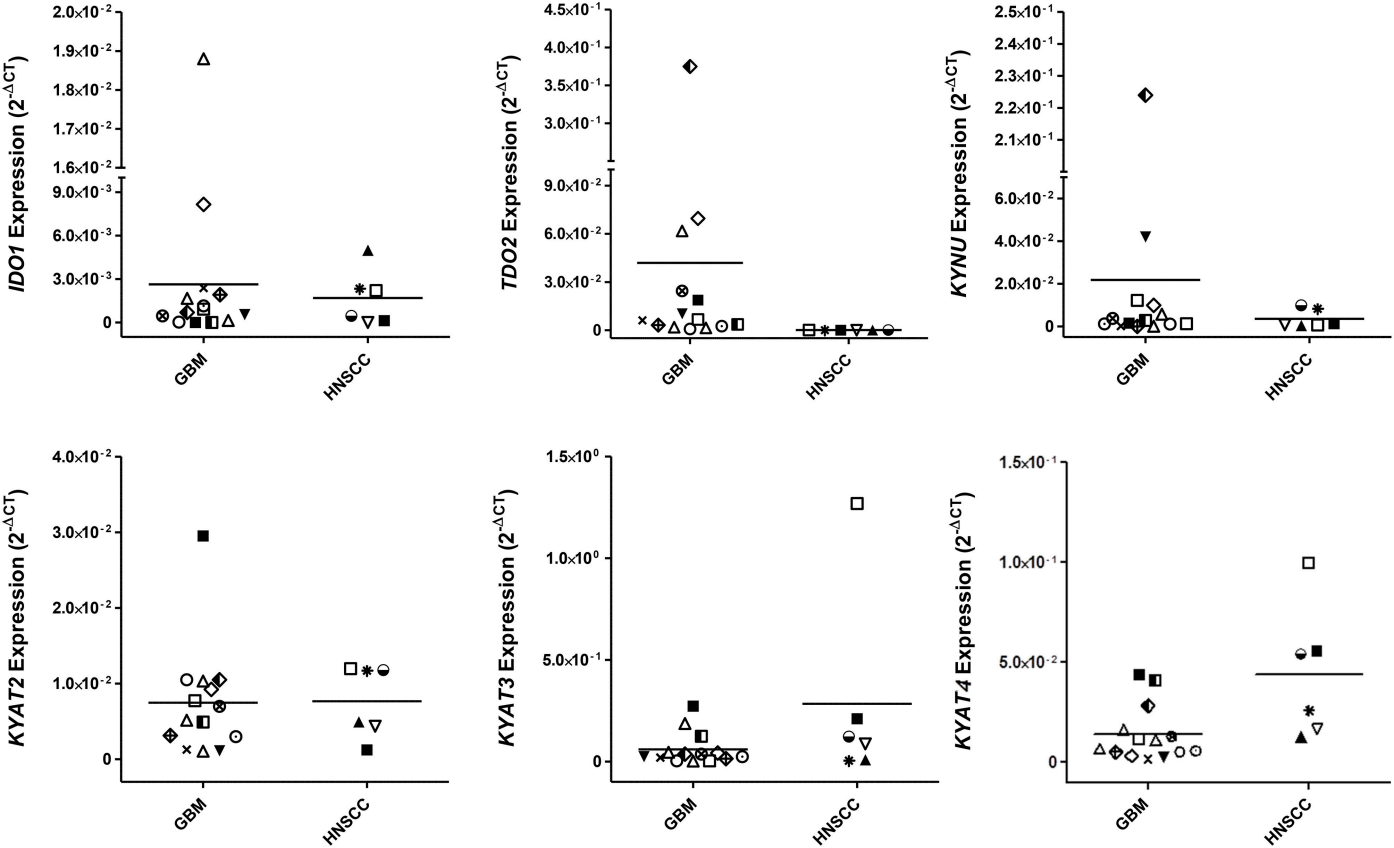
Relative messenger RNA (mRNA) expression of IDO1, TDO2, KYNU, KYAT2, KYAT3, and KYAT4 in glioblastoma multiforme (GBM) and head and neck squamous cell carcinoma cells (HNSCC). The graphs indicate the mRNA expression normalized to the housekeeping genes (2^−Δ^CT). GBM (*N* = 13, HROG02, HROG04, HROG05, HROG06, HROG10, HROG15, HROG24, HROG36, HROG38, HROG52, HROG63, HROG73, HROG75) and HNSCC cell lines [*N* = 6; FADU, Detroit-562, Cal-33, PE/CA/PJ-15, UT-SCC14, UT-SCC-15 (33)].

While IDO1 itself is not the only mechanism by which tumors can resist immune-mediated killing, we studied the expression status of different KP-related genes on a panel of human GBM and HNSCC cell lines. These experiments revealed not only differences between both entities but also a heterogeneous profile of all tested genes among cell lines (Figure 1). *IDO1* was differently expressed by most glioma samples (11/13) analyzed. In general, *IDO1* was only detectable at very low levels (Figure 1). *TDO2*, the other rate-limiting enzyme of the KP (36), was constitutively expressed by all glioma samples, and expression was even higher in comparison to *IDO1*. Generally, expression status for *TDO2* and kynurenine hydrolase (*KYNU*) was higher in GBM, while HNSCC expressed more kynurenine aminotransferases (*KYAT*) (Figure 1). Hence, these data indicate that the KP is active in both entities, with however different enzymes being involved in TRP catabolism.

**Figure 2:**
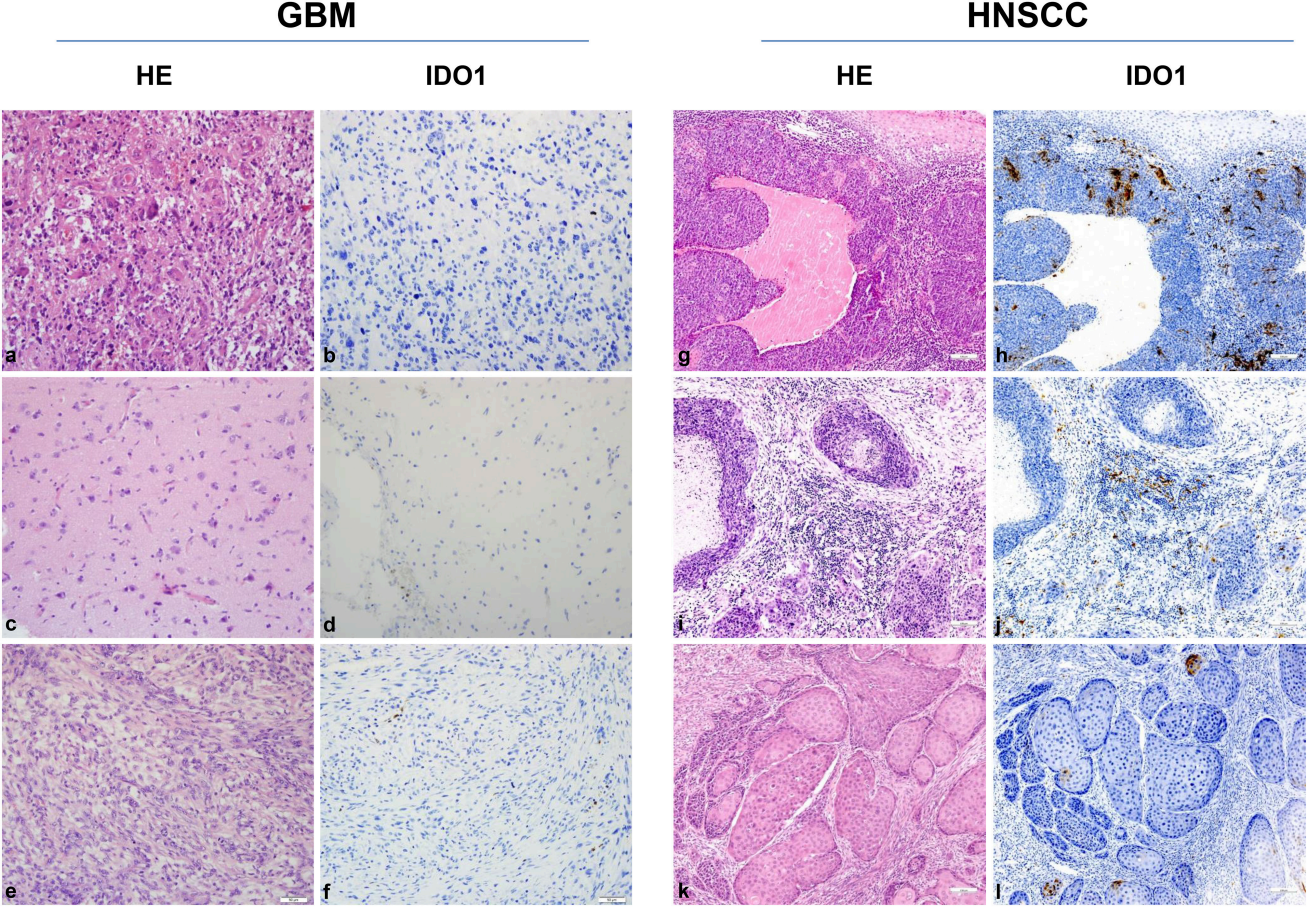
IDO1 immunohistochemistry. Representative images of primary glioblastoma multiforme (GBM) [HROG36 **(a,b)**, HROG63 **(c,d)**, and HROG73 **(e,f)**] and head and neck squamous cell carcinoma (HNSCC) [HNSCC06 **(g,h)**, HNSCC02 **(i,j)**, HNSCC01 **(k,l)**] samples. Left panel: Routine HE staining. Right panel: Note the focal IDO1 expression on tumor-infiltrating lymphocytes exclusively in HNSCC cases. HNSCC case 1 **(g,h)**: tonsil (HPV^positive^); case 2 **(i,j)**: mouth base (HPV^negative^, relapse); case 3 **(k,l)**: larynx (HPV^negative^). Pictures were taken at 20× and 10× magnification, respectively.

Still, tumor cell lines grown *in vitro* not necessarily represent the *in vivo* situation; we therefore analyzed the IDO1 abundance in clinical resection specimens (Figure 2). In GBM, IDO1 was detectable in one of three cases (representative images are shown in Figure 2). By contrast, HNSCC samples presented with IDO1 but only on a small fraction of tumor-infiltrating lymphocytes (Figure 2). Although not analyzed systematically, the only HPV^positive^ case in this small cohort showed highest IDO1 abundance, nicely reflecting the tumors' immunogenicity (11, 37).

### Gene Expression and Protein Changes Upon IFNγ Stimulation

IDO1 is an IFNγ-inducible enzyme. Upon stimulation, the KP is activated to induce immunosuppression. *In vitro* stimulation with IFNγ mimics the *in vivo* situation of an inflammatory microenvironment. Hence, upon immune-mediated inflammation, IDO1-negative tumor cells may upregulate *IDO1* as resistance mechanism.

**Figure 3:**
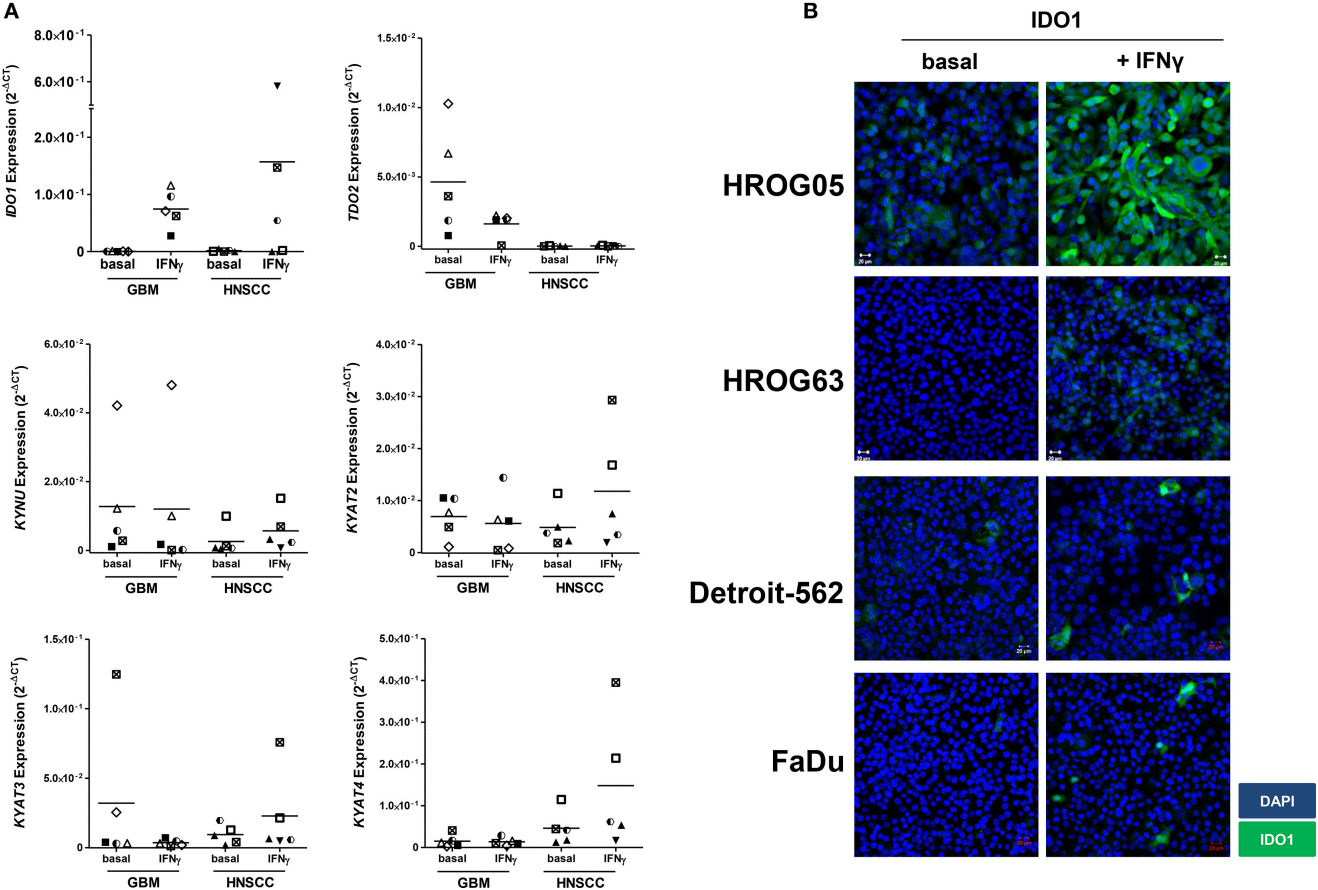
Relative messenger RNA (mRNA) expression of *IDO1, TDO2, KYNU, KYAT2, KYAT3*, and *KYAT4* as well as IDO1 protein abundance following interferon-γ (IFNγ) stimulation in glioblastoma multiforme (GBM) and head and neck squamous cell carcinoma (HNSCC) cells. The cell cultures were either untreated or treated with IFNγ (50 ng/ml) for 24 h. **(A)** The graphs indicate the mRNA expression normalized to the housekeeping genes (2^−Δ^CT). GBM *N* = 5 cell lines (HROG02, HROG05, HROG52, HROG63, HROG75); HNSCC, *N* = 4 cell lines (FADU, Detroit-562, Cal-33, PE/CA/PJ-15). **(B)** IDO1 immunofluorescence in selected cell lines. Cell nuclei were stained with DAPI, and IDO1 was depicted by monoclonal rabbit IDO1 primary antibody (1:100; Cell Signaling Technology), followed by secondary antibody (1:250, Boster Biological Technology Pleasanton, CA, United States) labeling. Cells were analyzed with a Zeiss LSM-780 Confocal Laser Microscope. Original magnification 20×.

Using five individual GBM cell lines, *IDO1* expression was inducible in all cases (Figure 3A). Upregulation of *IDO1* was high on protein levels in HROG05 cells and marginal in HROG63 (Figure 3B). *TDO2* and *KYAT3* were suppressed upon IFNγ stimulation in three of five samples and hardly detectable in one cell line, supporting data from a recent publication (38). *KYNU* was not affected by IFNγ stimulation (Figure 3A).

Just as in GBM, *IDO1* was inducible in HNSCC cells (Figure 3A). Immunofluorescence revealed focal expression of singular cells with different intensity (Figure 3B). Of note, IFNγ stimulation even induced upregulation of *KYAT1, KYAT2, KYAT3*, and *KYAT4* (Figure 3A and data not shown), most likely constituting a compensatory mechanism as described before in experimental autochthonous tumor models (39).

### Interference With the KP of Cytostatic and Targeted Therapies

Next, we examined whether cytostatic and targeted drugs have an influence on the KP. For GBM, TMZ was chosen, and for HNSCC, 5-FU, Cisplatin, Gemcitabine, as well as Cetuximab were used. As a targeted yet still experimental agent, the potent and specific CDKi Dinaciclib was applied to cells of both entities.

Before this experiment, drug doses were carefully tested in dose-response analyses (data not shown) along with discrimination of apoptosis and necrosis. Generally, drugs used in this study tended to induce necrosis, while apoptosis, if present, was only detectable at early time points. Exemplary results for the HNSCC cell line Detroit-562 are given in Supplementary Figures 1A,B. While cytostatics are well-known to affect normal cells' viability, the impact of the CDKi Dinaciclib on immune and red blood cells is less clear. We therefore performed a hemolysis and leukocyte viability assay. In this experiment, no toxicity was seen against normal cells (Supplementary Figure 1C). Even at high concentrations, Dinaciclib impaired cellular viability/integrity only marginally (Supplementary Figure 1C).

**Figure 4:**
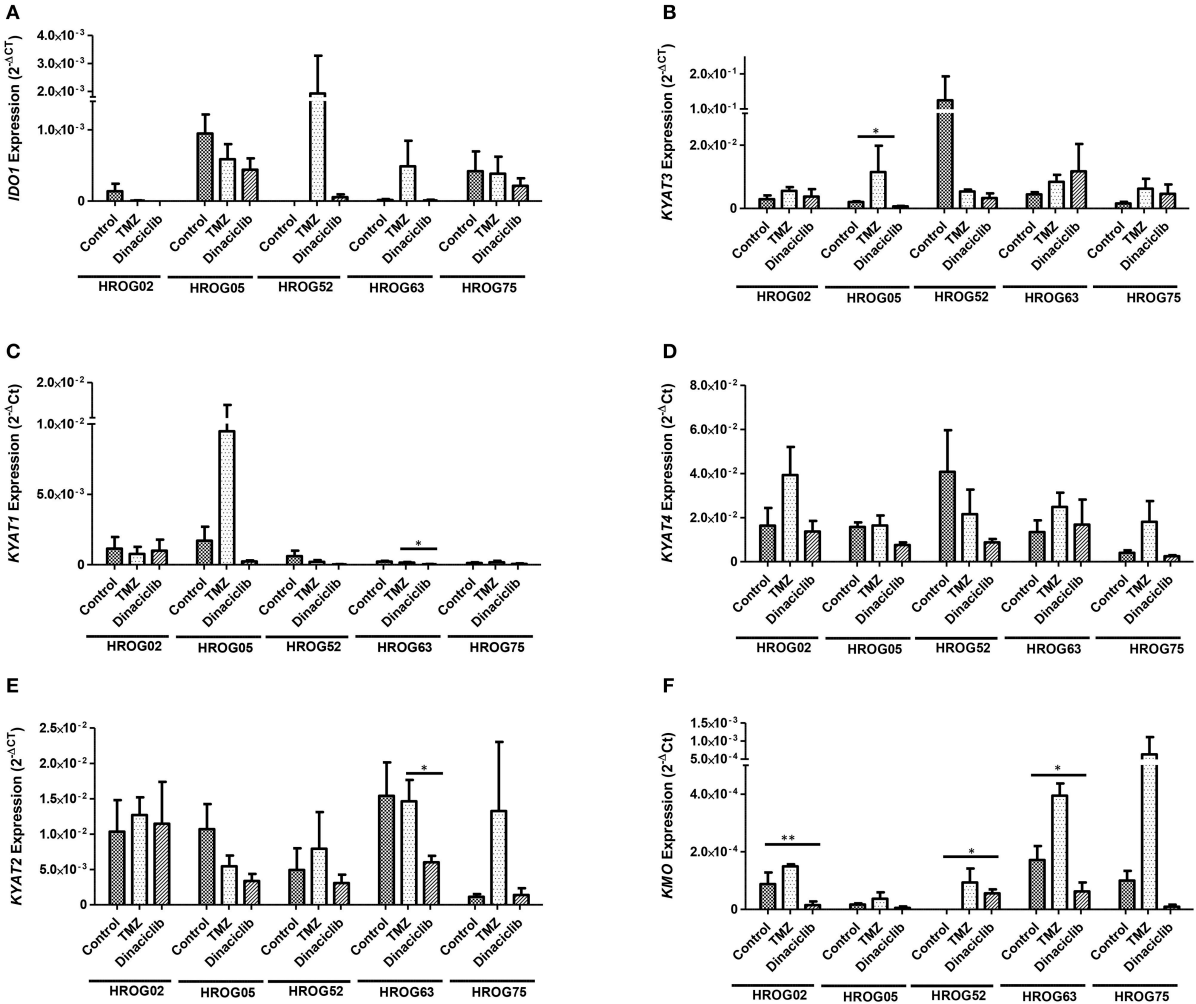
Relative messenger RNA (mRNA) expression of *IDO1, KYAT1, KYAT2, KYAT3, KYAT4*, and KMO upon cytostatic drugs and targeted therapy in glioblastoma multiforme (GBM) cells. Graphs indicate the mRNA expression of selected kynurenine pathway (KP)-related genes normalized to the housekeeping genes (2^−Δ^CT). Results show data of three independent experiments. *p < 0.05; **p < 0.01 vs. control. Two-way ANOVA.

**Figure 5:**
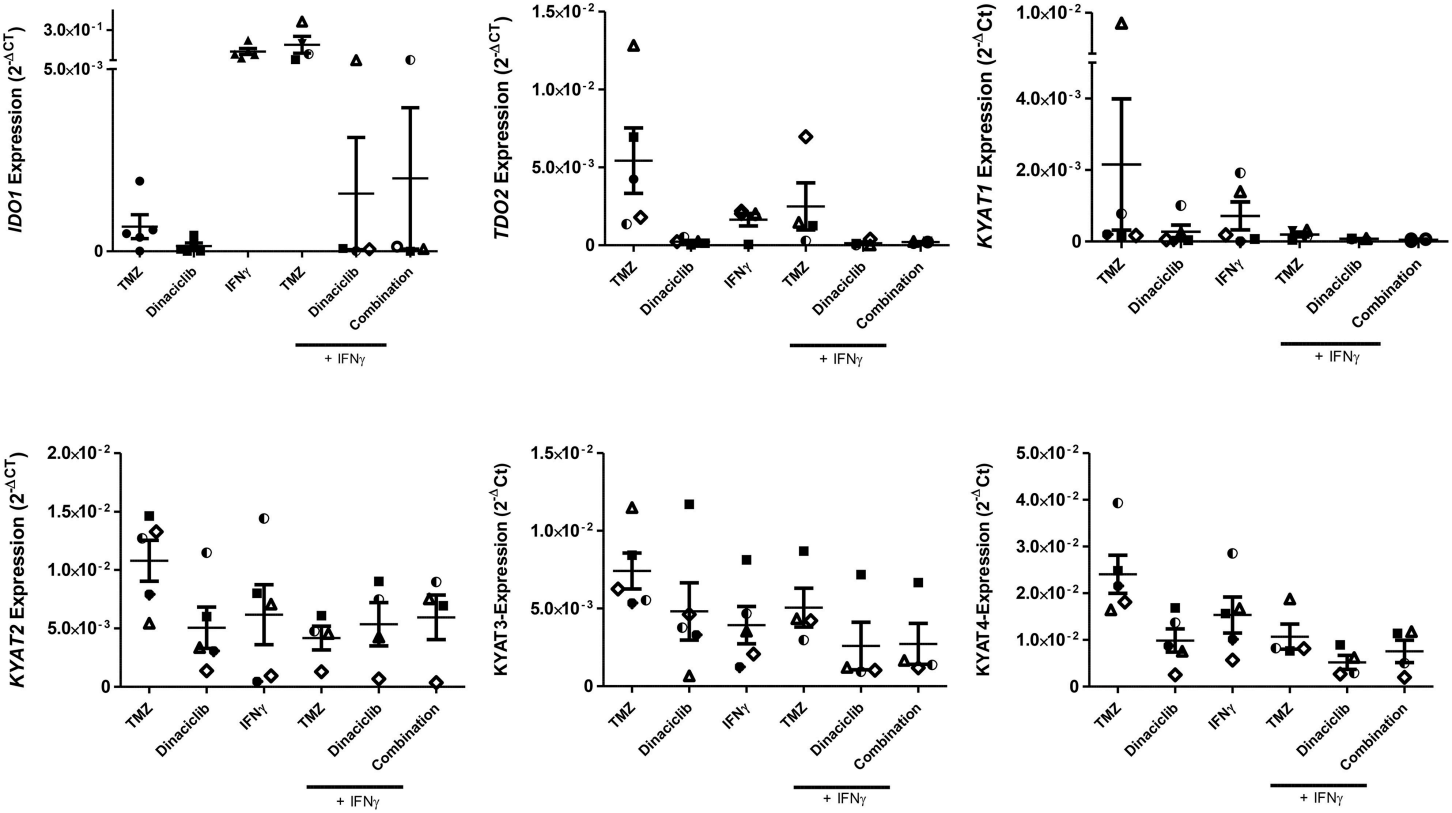
Relative messenger RNA (mRNA) expression of *IDO1, TDO2, KYAT1, KYAT2, KYAT3*, and *KYAT4* upon concomitant treatment with interferon-γ (IFNγ) and cytostatic drugs in glioblastoma multiforme (GBM) cells. The cell cultures (*N* = 5, HROG02, HROG05, HROG52, HROG63, HROG75) were either left untreated or treated with IFNγ (50 ng/ml) for 24 h. Treatments were performed simultaneously, i.e., IFNγ ± TMZ, and/or Dinaciclib. Graphs indicate the mRNA expression of selected KP-related genes normalized to the housekeeping genes (2^−Δ^CT). Results show data of three independent experiments. A statistical report is given in Supplementary Table for Figure 5.

TMZ is an oral alkylating agent that methylates DNA at the O^6^ position of guanine causing cell cycle arrest at G2/M. It is used as standard of care for GBM. However, acquired resistance, a process not fully understood, leads to major limitations in treatment. Here, TMZ downregulated *IDO1* in three of five GBM cell lines but led to increased expression in HROG52 and HROG63—a paired GBM cell line established from the very same patient (primary lesion and upon relapse) (Figure 4). Gene expression of *KYAT2, KYAT4*, and *KMO* was heterogeneous. Generally, there was a trend toward higher expression of those genes but with cell-line-specific differences (e.g., *KYAT3*: *p* < 0.05 vs. control in HROG05 cells; Figure 4). *KYNU* expression was not affected by TMZ (data not shown). Interestingly, the combination of IFNγ and TMZ that mimics the *in vivo* situation led to similar or even stronger *IDO1* upregulation compared to IFNγ alone in two out of four glioma samples (Figure 5). Adding Dinaciclib to either IFNγ or TMZ lowered the mRNA expression of *IDO1* massively. Other KP-related genes like *TDO2* and *KYAT1-4* were similarly downregulated (Figure 5). Supplementary Table for Figure 5 provides a detailed statistical analysis of each cell line in relation to the individual treatment regimens.

**Figure 6:**
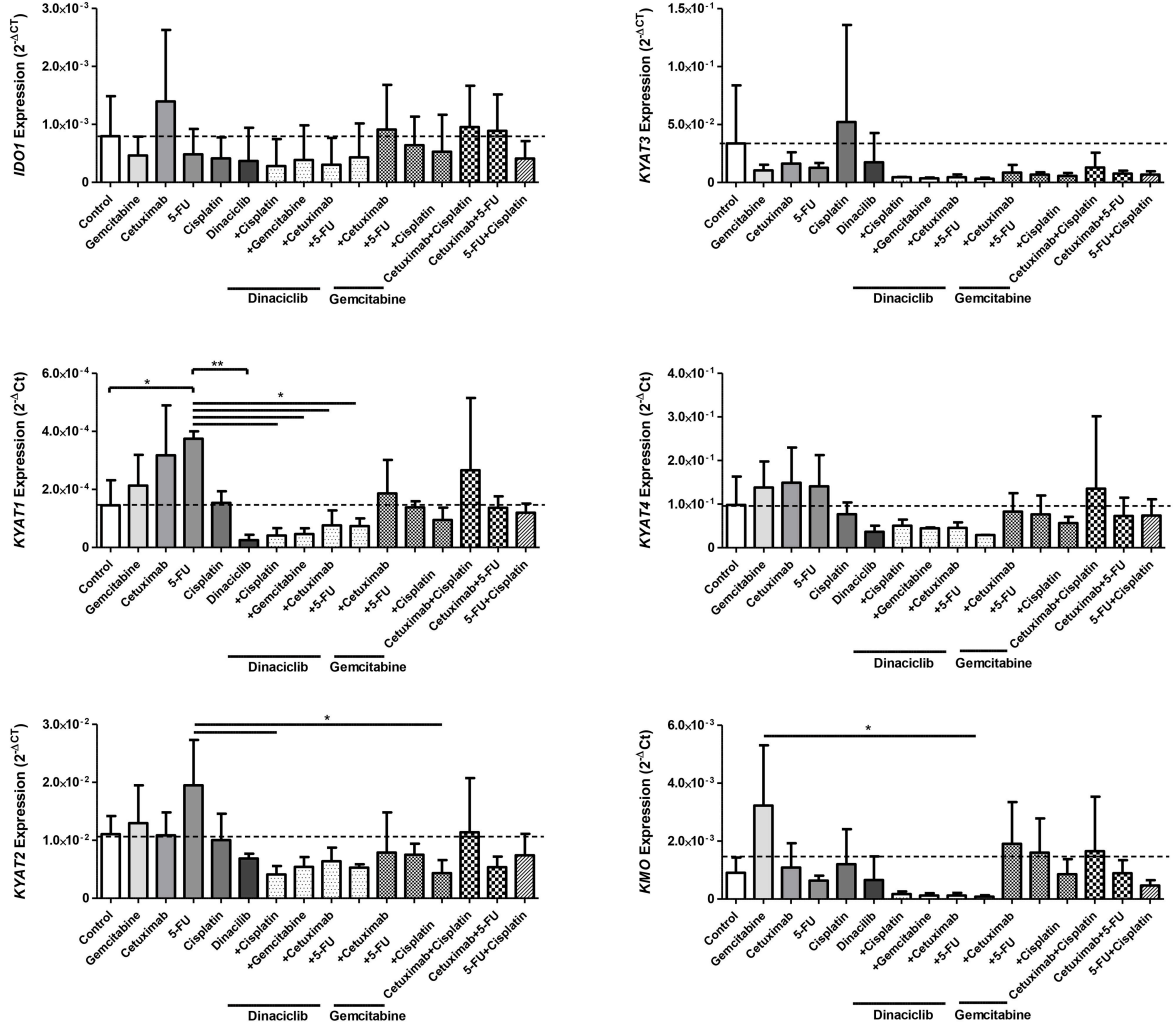
Relative messenger RNA (mRNA) expression of *IDO1, KYAT1, KYAT2, KYAT3, KYAT4*, and KMO upon cytostatic drugs and targeted therapy in head and neck squamous cell carcinoma (HNSCC) cells. Graphs indicate the mRNA expression of selected KP-related genes normalized to the housekeeping genes (2^−Δ^CT). Results show data of three independent experiments using HNSCC cell line Detroit-562. *p < 0.05; **p < 0.01. One-way ANOVA (Bonferroni's multiple comparison test).

In HNSCC cells, Cetuximab was the only *IDO1*-inducing substance (exemplary results for Detroit-562 cells are given in Figure 6). Beyond that, the cytostatics as well as Cetuximab induced at least one of the KP-related genes (*p* < 0.05 vs. control), implicating activation of this pathway *via* different effectors. By adding Dinaciclib to cytostatic drugs, this effect was abrogated, even in the presence of IFNγ (Figure 6 and data not shown). Of note, Dinaciclib alone as well as in combination with other substances effectively suppressed all KP-related genes, implying inhibition of the TRP catabolism by this CDKi.

### Dinaciclib Blocks IFNγ-Induced IDO1 Expression in GBM and HNSCC Cells

Considering the active downregulation of KP-related genes by Dinaciclib, we investigated whether this CDKi is able to inhibit or reverse IFNγ-induced IDO1 upregulation in GBM and HNSCC cells on a protein level. TMZ and Cetuximab were included as active inductors of *IDO1* and associated KP-related genes.

**Figure 7:**
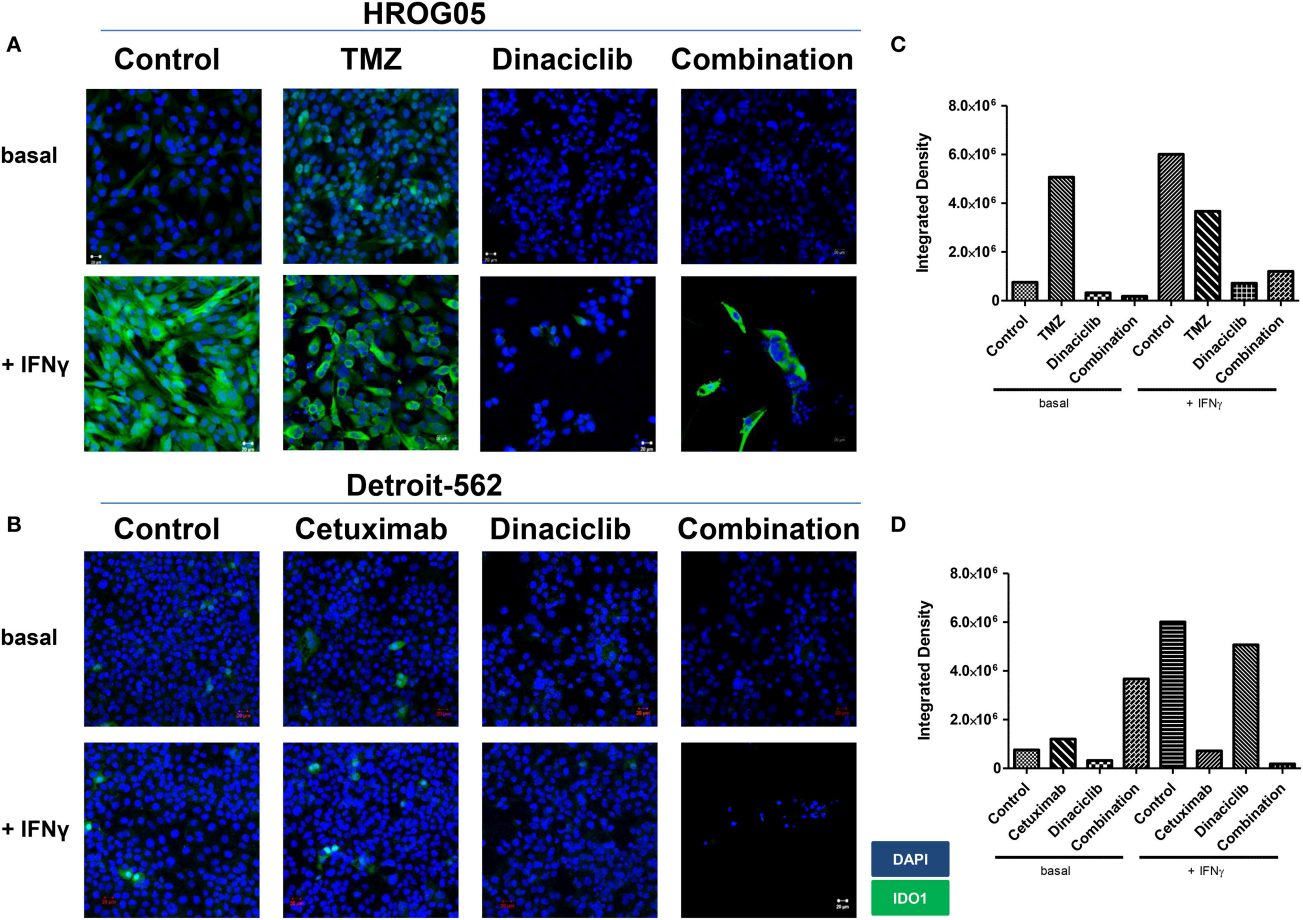
Indoleamine 2,3-dioxygenase (IDO1) protein abundance in selected glioblastoma multiforme (GBM) and head and neck squamous cell carcinoma (HNSCC) cells upon cytostatic drugs and targeted therapy. The cell cultures [**(A)** GBM: HROG05; **(B)** HNSCC: Detroit-562] were either left untreated or treated with IFNγ (50 ng/ml) for 24 h. Treatments were performed simultaneously, i.e., IFNγ ± TMZ, Cetuximab, and/or Dinaciclib. Cell nuclei were stained with DAPI. Original magnification 20×. **(C,D)** Quantification was done to score staining intensity in untreated and treated HROG05 and Detroit-562 cells. This was carried out using ImageJ software as described in Material and Methods.

IFNγ and selected drugs were added simultaneously for 72 h. Dinaciclib effectively blocked IFNγ-induced IDO1 protein in both entities, while TMZ alone as well as the combination with IFNγ strongly enhanced IDO1 protein level (Figure 7). Hence, mRNA expression data were nicely confirmed.

When Dinaciclib was combined with IFNγ and TMZ, the IDO1-inducing stimulus of these latter substances was far too strong to be suppressed (Figure 7). However, the low number of residual cells in this combination hints toward additive or even synergistic effects independent from IDO1 (Figures 7A,B).

While IDO1 was highly inducible in GBM cells only, we then determined protein level upon IFNγ-prestimulation approaching the *in vivo* situation. The cytotoxic effect of Dinaciclib was preserved; however, levels of IDO1 enzyme were not significantly altered (Supplementary Figures 2A,B). Comparable results were obtained for TMZ. Virtually, all residual cells showed positive staining; still there was a trend toward lower intensity in monotherapy and in combination (Supplementary Figure 2B).

Taken together, the CDKi Dinaciclib is able to block IFNγ-mediated and thus most likely even chemotherapy-induced *IDO1* upregulation in GBM and HNSCC cells. However, blunt interference with this TRP-metabolizing enzyme is unlikely.

### Treatment Induced Influence on KP-Related Metabolites

Our data revealed IDO1 induction by TMZ, which is reversible by Dinaciclib. Thus, we examined the influence on KP-related metabolites in GBM cell lines.

**Figure 8:**
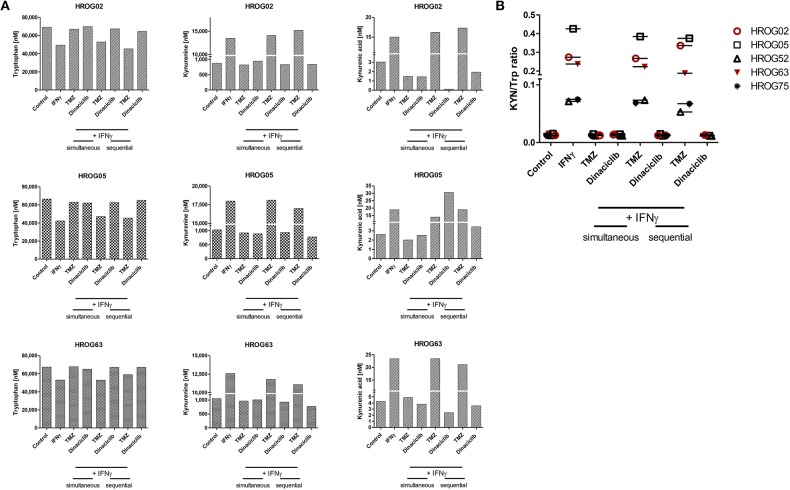
Indoleamine 2,3-dioxygenase (IDO) protein abundance in glioblastoma multiforme (GBM) and head and neck squamous cell carcinoma (HNSCC) cells as well as kynurenine pathway (KP) metabolite levels in GBM cells upon cytostatic drugs and targeted therapy. Treatments were performed sequentially, i.e., interferon-γ (IFNγ) pretreatment for IDO1 induction, followed by Temozolomide (TMZ) and/or Dinaciclib. **(A)** KP metabolites changed upon TMZ but not Dinaciclib treatment. The combination of TMZ and IFNγ accelerates tryptophan (TRP) consumption accompanied by kynurenine (KYN) and kynurenic acid (KYNA) production **(B)**. KYN/TRP ratios in GBM cells were determined dividing KYN values by TRP values. Results show data of a single measurement.

TRP, KYN, and the downstream metabolite kynurenic acid (KYNA) were quantified by MS using cell culture supernatants of GBM cell lines (Figures 8A,B). TRP was catabolized after 24 h from all cell lines among all treatment regimens. Adding TMZ or Dinaciclib in monotherapy marginally affected TRP consumption as well as KYN and KYNA production. Stimulation with either IFNγ or a combination of TMZ resulted in greatly enhanced TRP depletion and increased KYN levels, although to varying degrees in the different cell lines (Figure 8A). Small amounts of KYNA were produced constitutively and to a greater extent after IFNγ mono- and TMZ combination in all cell lines (Figure 8A). In contrast, KYNA level remained unchanged upon Dinaciclib in combination with IFNγ, confirming immunofluorescence results (please see Figure 7 for details). The same was true for the KYN/TRP ratio, being only affected in samples treated with IFNγ as well as the combination of IFNγ and TMZ (Figure 8B).

These data underline our gene and protein expression data. The CDKi Dinaciclib is directly or indirectly capable of blocking the KP. TMZ particularly in combination with the proinflammatory cytokine IFNγ accelerates TRP consumption accompanied by KYN and KYNA production in GBM cells.

## Discussion

The finding that high *IDO1* expression is associated with shorter survival in cancer patients made IDO1 a promising target either by specific inhibitors or indirectly by immunomodulation. A recent study described dramatically suppressed tumor growth upon *IDO1* knockdown by increasing the number of CD4^+^ and CD8^+^ T cells in murine GBM models (9). However, the exact mechanisms underlying IDO1 and thus TRP metabolism along the KP remain unclear. Therefore, we focused on the expression of *IDO1* and *IDO*-related KP genes and their potential involvement in immune evasion in experimental models of HNSCC and GBM.

We were able to show that the KP is active in both entities, with different enzymes involved in TRP catabolism. Of note, basal *IDO1* expression was low and inversely correlated with *TDO2*. In the only prior study on primary GBM cultures, similar results were described with constitutive *TDO2* expression in most GBM cell cultures (29). In here, TDO2 likely promotes tumor growth by suppressing antitumor immune responses (2, 31). KP products are considered as therapeutic targets because *IDO1* and other genes of the TRP metabolism are not expressed in healthy brain tissue, but gradually increase with GBM dedifferentiation (low vs. high grade GBM). In HNSCC, different results on *IDO1* are documented, and expression is heterogeneous among different HNSCC cell lines. Of note, IDO1 abundance of primary resection specimen and cultured cells seems to be independent from anatomical site and HPV status (40). Still, IDO1 is a useful marker for progression of in oral squamous cell carcinoma (41). In esophageal squamous cell carcinoma, progression and metastasis correlates with strong inflammation at the tumors' invasive front and disturbed TRP metabolism (42). These cumulative data highlight the biological relevance of the KP in malignancies and may explain why IDO1 is barely detectable upon long-term *in vitro* culture. By mimicking the inflamed microenvironment and thus taking a step closer to the *in vivo* situation, IFNγ was added as strong IDO1 inductor (43). While GBM cells responded with the expected *IDO1* upregulation on mRNA expression and protein level as well as accelerated TRP consumption, this molecule was barely inducible in HNSCC cells. It is conceivable that this is due to the duration of *in vitro* culture. GBM cells were established recently and thus used in defined low passages (<P40), whereas half of the HNSCC cell lines were long-term cultures with more or less unknown passage [Detroit-562 as well as UT-SCC14 and UT-SCC15 (44) are the only exceptions; <P40]. Cell lines may acquire additional mutations overtime changing their protein expression. Another *in vitro* limitation is that experiments were conducted without immunological pressure. *In vivo* studies are desirable to verify the results.

Indirect effects of TRP metabolism include interference with other biological functions like migration, angiogenesis, and cell growth regulation (18, 40). To investigate the influence of anticancer drugs on TRP catabolism, we performed a comprehensive analysis using conventional chemotherapy (TMZ, 5-FU, Cisplatin, Gemcitabine) and targeted drugs (Cetuximab, Dinaciclib). The KP-related gene expression and metabolites were determined in residual cells. In GBM, the standard of care drug TMZ was applied either with or without IFNγ stimulation. While this substance affected *IDO1* on the expression level, the amount of the resulting protein increased. This may be explained by either increased protein's half-life due to a reduced rate of degradation or the preferential translation during cellular stress. In previous studies, exposure of several cultured human malignant glioma cell lines, primary neurons, and a neuroblastoma cell line to IFNγ reduced TRP levels in culture medium accompanied by increased *IDO1* expression and KYN production (29, 45). Our results confirm these data, and in addition, we were able to demonstrate that IFNγ stimulation in combination with TMZ stimulated KYN and KYNA production and TRP catabolism in GBM cell cultures. The increase in TRP catabolism and KYN production (KYN/TRP ratio) is widely used as indirect indicator of the cumulative activities of TDO2, IDO1, and IDO-2 (38, 46). The KP in brain tumors is likely triggered by IFNγ from immediate surrounding tissue (29, 47, 48). Thus, *IDO1* expression in brain tumor cells is likely to be triggered when IFNγ is produced from activated T cells and/or microglia and neurons. Furthermore, gliomas and glioneuronal tumors have an elevated tryptophan uptake and catabolism *in vivo* (49). Given our observation on a further enhanced KP activity upon TMZ treatment, this might provide an explanation of (acquired) drug resistance and final relapse. Hence, IDO1 blocking agents should be investigated in TMZ-tailored therapeutic approaches.

In HNSCC cells, KP activation was different. KP-related genes were exclusively induced by standard drugs, and only Cetuximab induced *IDO1*. Additional upregulated genes involved kynurenine aminotransferases, responsible for synthesizing a neuroprotectant, and KMO. While the specific biochemical activity of these molecules and biological relevance in cancer is barely examined, we interpret this result as one possible mechanism of resistance upon therapy—a finding quite common after conventional chemotherapy and usually also being associated with poor response toward neoadjuvant therapy in other entities (50).

Mechanistically, this can be attributed to the secretion of proinflammatory substances, such as prostaglandin E2 or high-mobility group protein B1 by dying tumor cells, secondary contributing to KP activation. By accumulating TRP, toxic metabolites of tumor cells actively shape an immunosuppressive microenvironment. Breaking down this shield is one of the main objectives in pharmacological inhibition of KP. Questions remain why most inhibitors failed in clinical trials, and mechanisms are only just beginning to become clear. A fact worth mentioning is the functional redundancy of IDO1, IDO-2, and TDO2 (51), augmenting the risk of mechanistic bypass.

Dinaciclib is a potent and specific CDK inhibitor of CDK1, CDK2, CDK5, and CDK9. Preclinical studies showed that this inhibitor is capable of decelerating tumor growth in numerous cancer entities via cell cycle arrest and apoptosis induction (52, 53). In our study, Dinaciclib was the only KP-inhibiting substance tested here. Of note, impairment of the KP was independent from the combination partner, and this CDKi effectively suppressed IFNγ-induced *IDO1* upregulation after simultaneous treatment. While this result was completely unexpected and has—to the best of our knowledge—not been described previously, our data do not support the idea of blunt interference with the KP. GBM cells with strong *IDO1* expression showed only marginally reduced IDO1 protein level after Dinaciclib treatment. This effect might be boosted after long or repeated treatment cycles. In line with these findings, several preclinical studies already proposed synergistic effects of selective and unselective IDO1 inhibitors when administered in conjunction with chemo- and/or radiotherapy (4). This may finally have impact for second- or third-line immunotherapeutic approaches. Therefore, the late KYN/TRP index is indeed a relevant clinical benchmark providing prognostic value for GBM patients (54).

Summarizing our findings, we provide evidence for the relevance of TRP catabolism in malignancies especially in the context of standard therapy. The CDKi Dinaciclib was identified as indirect KP inhibitor. Lastly, specific KP inhibition may increase the efficacy of standard drugs by restoring immune function and thus improve patients' outcome.

## Data Availability Statement

The datasets generated for this study are available on request to the corresponding author.

## Author Contributions

CR performed experiments, analyzed data, and participated in manuscript writing. BS and AZ performed immunohistochemistry and analysis, and provided images. HK, JG, NI, and FS performed experiments and analyzed data. GD performed LC-MS analyses. CC and CJ participated in paper finalization and critically revised the manuscript. DS and EW critically revised the manuscript. CM designed study, the outline of the manuscript, performed data interpretation, and wrote the manuscript.

### Conflict of Interest

The authors declare that the research was conducted in the absence of any commercial or financial relationships that could be construed as a potential conflict of interest.

## Supplementary Material

Supplementary Figure 1 Quantitative analysis of cell death in Detroit-562 HNSCC cells upon cytostatic drugs and targeted therapy. The cells were treated with the given substances for 24 and 72 h. Thereafter, cells were harvested and stained with Yo-Pro-1 to detect early and late apoptotic cells, as well as propidium iodide for necrosis determination. Apoptosis/necrosis discrimination was done on a flow cytometer (BD FACSVerse^TM^) as described in material and methods. **(A)** Quantitative analysis of cell death after 24 and 72 h, respectively. ^*^*p* < 0.05 vs. control; ^**^*p* < 0.01 vs. control. *t*-test. **(B)** Representative dot plots showing elevated numbers of necrotic cells upon treatment. **(C)** Hemolysis and viability of PBMC upon treatment with Dinaciclib. Therefore, whole blood and PBMC were cultured in the presence of increasing Dinaciclib concentrations (1, 5, and 10 μM) for 2 and 24 h, respectively. Hemolytic activity was determined from cell-free supernatants (red blood cell lysis), Calcein AM was used for quantifying viability of PBMC. Mean + SD, *N* = 5 individual donors.

Supplementary Figure 2 IDO1 protein abundance in HROG05 GBM cells upon cytostatic drugs and targeted therapy. The cells were pretreated with IFNγ (50 ng/ml) for 24 h. Thereafter TMZ, Dinaciclib and the combination of both substances was added to see whether IFNγ-induced upregulation of IDO1 is reversible. **(A)** None of these substances downregulated IDO1 in the sequential setting. Cell nuclei were stained with DAPI. Original magnification 20x. **(B)** Quantification was done to score staining intensity in untreated and treated HROG05 cells. This was carried out by using ImageJ software as described in material and methods.

Supplementary Table for Figure 5 Statistical analysis of individual treatment regimens, depicted for each cell line, and genes analyzed.

## Abbreviations

CDKi: cyclin-dependent kinase inhibitor
GBM: glioblastoma multiforme
HNSCC: head and neck squamous cell carcinoma
IDO1: indoleamine 2,3-dioxygenase
IFN: interferon
KYAT: kynurenine aminotransferase
KP: kynurenine pathway
PBMC: peripheral blood mononuclear cells
SCC: squamous cell carcinoma
TDO2: tryptophan 2,3-dioxygenase.
