# Supplementary material for: Activation of the Kynurenine Pathway in Human Malignancies Can Be Suppressed by the Cyclin-Dependent Kinase Inhibitor Dinaciclib
Source: Front Immunol. 2020 Feb 14;11:55. doi: 10.3389/fimmu.2020.00055 (PMC7034242; doi:10.3389/fimmu.2020.00055)
Supplement: Supplementary Figure 1 — Quantitative analysis of cell death in Detroit-562 HNSCC cells upon cytostatic drugs and targeted therapy. The cells were treated with the given substances for 24 and 72 h. Thereafter, cells were harvested and stained with Yo-Pro-1 to detect early and late apoptotic cells, as well as propidium iodide for necrosis determination. Apoptosis/necrosis discrimination was done on a flow cytometer (BD FACSVerseTM) as described in material and methods. (A) Quantitative analysis of cell death after 24 and 72 h, respectively. *p < 0.05 vs. control; **p < 0.01 vs. control. t-test. (B) Representative dot plots showing elevated numbers of necrotic cells upon treatment. (C) Hemolysis and viability of PBMC upon treatment with Dinaciclib. Therefore, whole blood and PBMC were cultured in the presence of increasing Dinaciclib concentrations (1, 5, and 10 μM) for 2 and 24 h, respectively. Hemolytic activity was determined from cell-free supernatants (red blood cell lysis), Calcein AM was used for quantifying viability of PBMC. Mean + SD, N = 5 individual donors. [file Presentation_1.PDF]

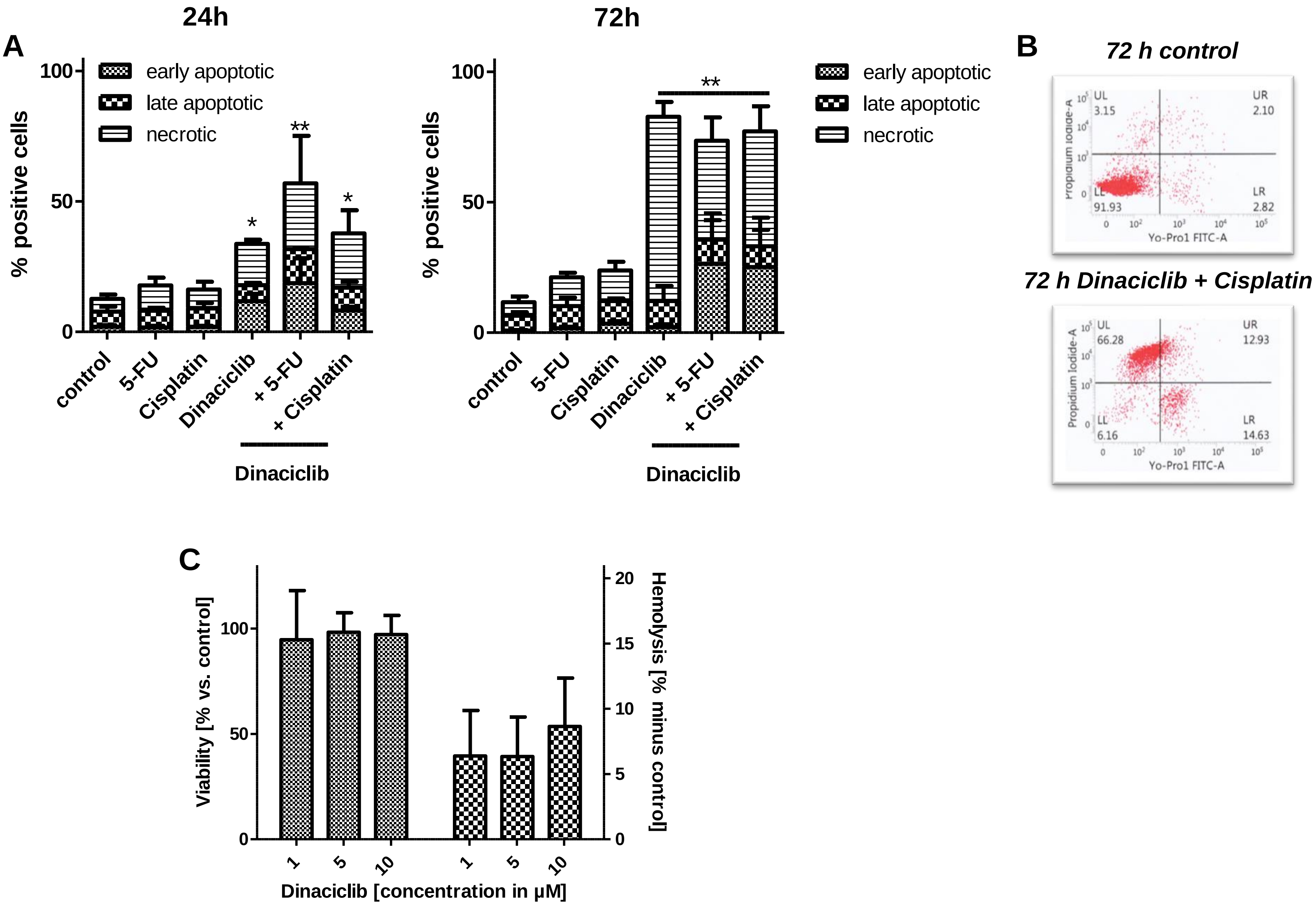

Supplementary Figure 1

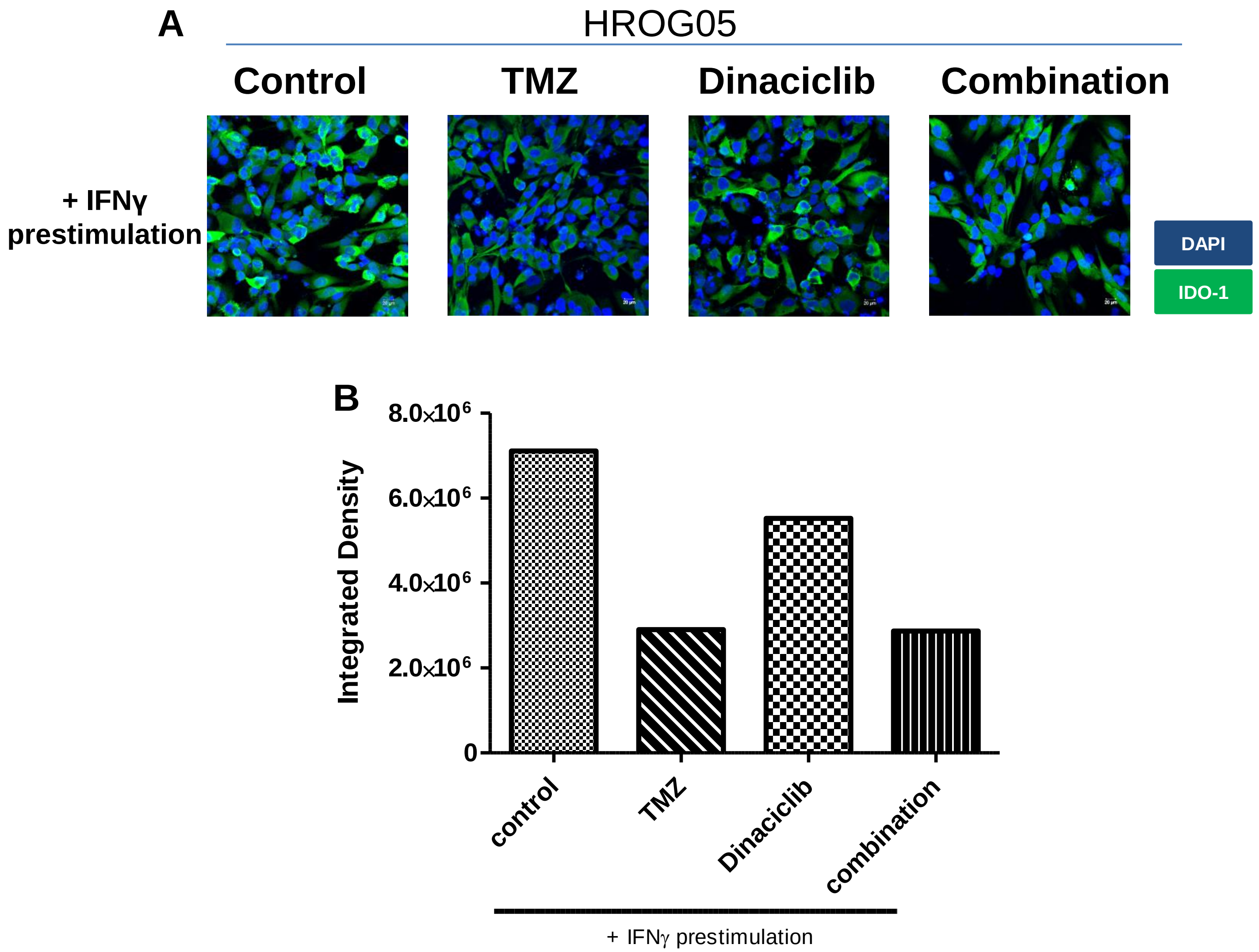

Supplementary Figure 2
