# Supplementary material for: Activation of the Kynurenine Pathway in Human Malignancies Can Be Suppressed by the Cyclin-Dependent Kinase Inhibitor Dinaciclib
Source: Front Immunol. 2020 Feb 14;11:55. doi: 10.3389/fimmu.2020.00055 (PMC7034242; doi:10.3389/fimmu.2020.00055)
Supplement: Supplementary Table for Figure 5 — Statistical analysis of individual treatment regimens, depicted for each cell line, and genes analyzed. [file Table_1.PDF]

Supplementary Table for Figure 5: Statistical analysis of invidual treatment regimens, depicted for each cell line and genes analyzed.

p-value	Gene name	Cell line	Condition
*p<0.05	*IDO*	HROG02	IFNγ vs. Dinaciclib + IFNγ, Dinaciclib + IFNγ vs. TMZ + IFNγ
		HROG05	Dinaciclib vs. IFNγ, TMZ vs. IFNγ
		HROG63	TMZ vs. IFNγ, Dinaciclib vs. IFNγ
		HROG75	basal vs. TMZ vs. IFNγ
	TDO	HROG02	Dinaciclib vs. IFNγ
		HROG05	basal vs. Dinaciclib, Dinaciclib vs. Dinaciclib + IFNγ, Dinaciclib + IFNγ vs. TMZ + IFNγ
		HROG63	IFNγ vs. Dinaciclib + IFNγ
		HROG75	basal vs. Dinaciclib, basal vs. IFNγ
	KMO	HROG02	TMZ vs. Dinaciclib + IFNγ
		HROG05	basal vs. TMZ + IFNγ, Dinaciclib vs. IFNγ, Dinaciclib vs. TMZ + IFNγ
		HROG52	basal vs. Dinaciclib
		HROG63	basal vs. TMZ, basal vs. TMZ + IFNγ,, Dinaciclib vs. IFNγ,<br>Dinaciclib vs. TMZ + IFNγ, TMZ vs. IFNγ, TMZ vs. Dinaciclib + IFNγ, TMZ vs. TMZ + IFNγ, IFNγ vs. Dinaciclib + IFNγ
		HROG75	basal vs. TMZ + IFNγ, Dinaciclib vs. TMZ + IFNγ
	KYNU	HROG02	Dinaciclib vs. TMZ + IFNγ, Dinaciclib + IFNγ vs. TMZ + IFNγ, Dinaciclib vs. IFNγ
		HROG05	basal vs. Dinaciclib, Dinaciclib vs. TMZ, Dinaciclib vs. IFNγ
		HROG63	Dinaciclib vs. IFNγ, TMZ vs. IFNγ
		HROG75	Dinaciclib vs. TMZ + IFNγ
	KYAT1	HROG63	Dinaciclib vs. TMZ
		HROG75	basal vs. TMZ vs. IFNγ, Dinaciclib + IFNγ vs. TMZ + IFNγ
	KYAT2	HROG63	Dinaciclib vs. TMZ
	KYAT3	HROG02	basal vs. TMZ + IFNγ, Dinaciclib + IFNγ vs. TMZ + IFNγ
		HROG05	basal vs. Dinaciclib, Dinaciclib vs. TMZ, Dinaciclib + IFNγ vs. TMZ + IFNγ
		HROG52	basal vs. Dinaciclib
		HROG75	Dinaciclib + IFNγ vs. TMZ + IFNγ
	KYAT4	HROG05	Dinaciclib vs. TMZ + IFNγ
		HROG75	Dinaciclib vs. TMZ + IFNγ
**p<0.01	*IDO*	HROG02	basal vs. IFNγ, basal vs. TMZ + IFNγ, Dinaciclib vs. TMZ + IFNγ, TMZ vs. IFNγ, TMZ vs. TMZ + IFNγ,<br>Dinaciclib vs. IFNγ
		HROG05	Dinaciclib vs. Dinaciclib + IFNγ, TMZ vs. Dinaciclib + IFNγ
	TDO	HROG02	IFNγ vs. Dinaciclib + IFNγ, IFNγ vs. TMZ + IFNγ,
		HROG05	Dinaciclib vs. TMZ + IFNγ, IFNγ vs. Dinaciclib + IFNγ
		HROG63	TMZ vs. Dinaciclib + IFNγ, Dinaciclib vs. TMZ, Dinaciclib vs. IFNγ
	KMO	HROG02	Dinaciclib vs. TMZ, TMZ vs. TMZ + IFNγ
		HROG63	basal vs. IFNγ, Dinaciclib vs. TMZ, Dinaciclib vs. IFNγ
	KYNU	HROG05	Dinaciclib vs. TMZ + IFNγ
	KYAT3	HROG02	basal vs. Dinaciclib + IFNγ
		HROG05	Dinaciclib vs. IFNγ
		HROG75	IFNγ vs. TMZ + IFNγ
	HAAO	HROG75	basal vs. Dinaciclib
***p<0.001	*IDO*	HROG63	Dinaciclib vs. TMZ + IFNγ, TMZ vs. TMZ + IFNγ, Dinaciclib + IFNγ vs. TMZ + IFNγ
	TDO	HROG05	Dinaciclib vs. IFNγ
	KYAT3	HROG05	Dinaciclib vs. TMZ + IFNγ
